# Ansa pancreatica identified on magnetic resonance cholangiopancreatography: a case report

**DOI:** 10.1093/jscr/rjaf1107

**Published:** 2026-01-20

**Authors:** Manisha Niure, Narendra Pandit, Aron Neupane, Bibisa Bhandari, Kshitiz Parajuli

**Affiliations:** Department of Gastrointestinal Surgery, Birat Medical College Teaching Hospital, Biratnagar 56613, Morang, Nepal; Department of Gastrointestinal Surgery, Birat Medical College Teaching Hospital, Biratnagar 56613, Morang, Nepal; Department of Gastrointestinal Surgery, Birat Medical College Teaching Hospital, Biratnagar 56613, Morang, Nepal; Department of Gastrointestinal Surgery, Birat Medical College Teaching Hospital, Biratnagar 56613, Morang, Nepal; Department of Gastrointestinal Surgery, Birat Medical College Teaching Hospital, Biratnagar 56613, Morang, Nepal

**Keywords:** ansa pancreatica, pancreatic duct anomalies, magnetic resonance cholangiopancreatography, young

## Abstract

A young female presented with acute epigastric pain radiating to the back, with associated symptoms of vomiting and a 2-year history of similar intermittent upper abdominal symptoms with no alcohol use history, smoking, or chronic illness. Laboratory investigations revealed elevated serum amylase and lipase, while imaging studies demonstrated acute-on-chronic pancreatitis with a dilated main pancreatic duct. Magnetic resonance cholangiopancreatography revealed a ductal anomaly, ansa pancreatica, characterized by a looping communication between the main and accessory pancreatic ducts draining via the minor papilla. Conservative management of symptoms continued to fail; therefore, a surgical procedure, Frey’s procedure, was performed, which showed improved clinical outcomes. This case highlights the clinical significance of ansa pancreatica as a rare but often misdiagnosed cause of recurrent pancreatitis in young patients without an alcohol use history.

## Introduction

Two or more episodes of acute pancreatitis that are fully resolved and in which the pancreatic enzymes return to normal in between episodes is acute recurrent pancreatitis (ARP) and is inherently a reversible clinical diagnosis. ARP with >3 episodes can lead to chronic pancreatitis, identified by duct dilation, pancreatic duct atrophy, or calcification [1].

Among the various etiologies of pancreatitis, alcohol remains the most common cause worldwide [2]. However, structural anomalies of the pancreatic duct are increasingly recognized as significant contributing factors, particularly in patients with idiopathic or recurrent forms of pancreatitis. Pancreas divisum is the most frequently identified ductal anomaly, while other variations, such as ansa pancreatica, are considerably rare and less well studied [3].

The communication between the Santorini duct and a branch of the Wirsung duct is imaged as a reversed S in Ansa pancreatica, originally described in 1961 in cadaveric studies. This has been proposed as a potential predisposing factor for pancreatitis [4–6].

This case report describes presentation of recurrent pancreatitis in conjunction with ansa pancreatica in a young female with no alcohol use history.

## Case discussion

A 19-year-old female presented to our emergency department with chief complaint of an insidious onset of epigastric pain for 1 day, progressive in nature, that radiated to her back. This pain typically worsened after meals and was relieved on bending forward.

It was associated with three episodes of vomiting, which were non-bilious, non-projectile, non-blood-stained, and contained undigested food particles.

She had a 2-year history of similar intermittent upper abdominal symptoms for which she had three hospital admissions to date. Her initial investigations from the first admission revealed progressive anemia, leukocytosis, and neutrophilia with elevated serum amylase. Raised alkaline phosphatase was also documented. An abdominal ultrasound revealed mild dilatation of the main pancreatic duct, leading to a suspicion of chronic pancreatitis, followed by multidetector computed tomography (MPCT) of the abdomen showing acute interstitial edematous pancreatitis superimposed on chronic pancreatitis. She was managed conservatively on all subsequent visits.

She denied a history of chronic alcohol consumption, smoking, and has no chronic conditions, and no prior history of surgical interventions. Her family history was non-contributory.

On examination, she appeared ill but was fully alert and oriented to time, place, and person with no signs of pallor, jaundice, clubbing, cyanosis, or edema. Her vitals were normal.

Abdominal examination revealed a soft and non-tender abdomen, with no palpable mass or organomegaly. Adequate bowel sounds were appreciated. The chest examination was unremarkable. There were no significant findings on neurological examination.

Patient’s lab findings showed red blood cell count of 4.03 (10^12^/l), a white blood cell count of 14 (10^9^/l), a serum amylase of 1331 U/l, and a serum lipase of 3020 U/l, while other tests, including liver function tests, kidney function tests, calcium, and HbA1c, were normal. The lipid profile indicated a cholesterol level of 118 mg/dl and lactate dehydrogenase (LDH) at 307 U/l.

**Figure 1:**
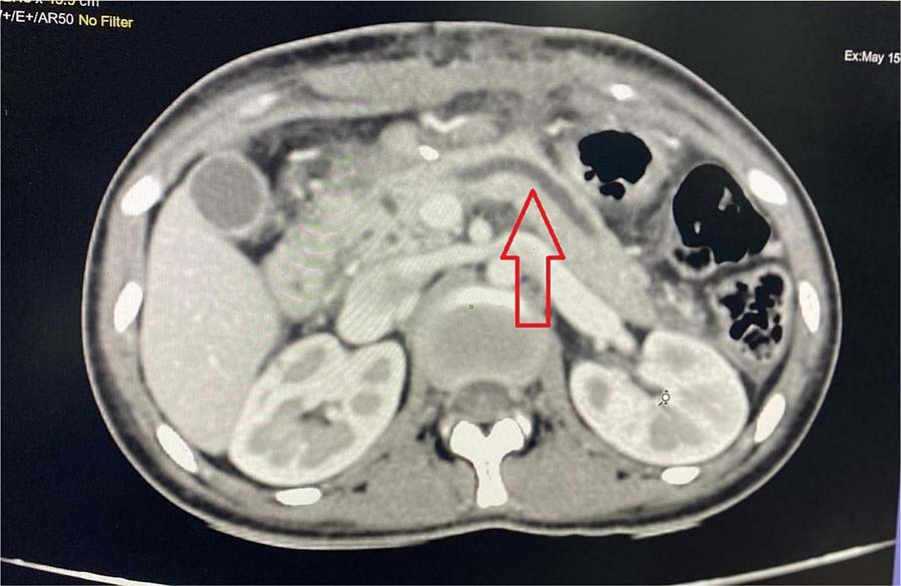
Abdominal CT showing dilated pancreatic duct.

**Figure 2:**
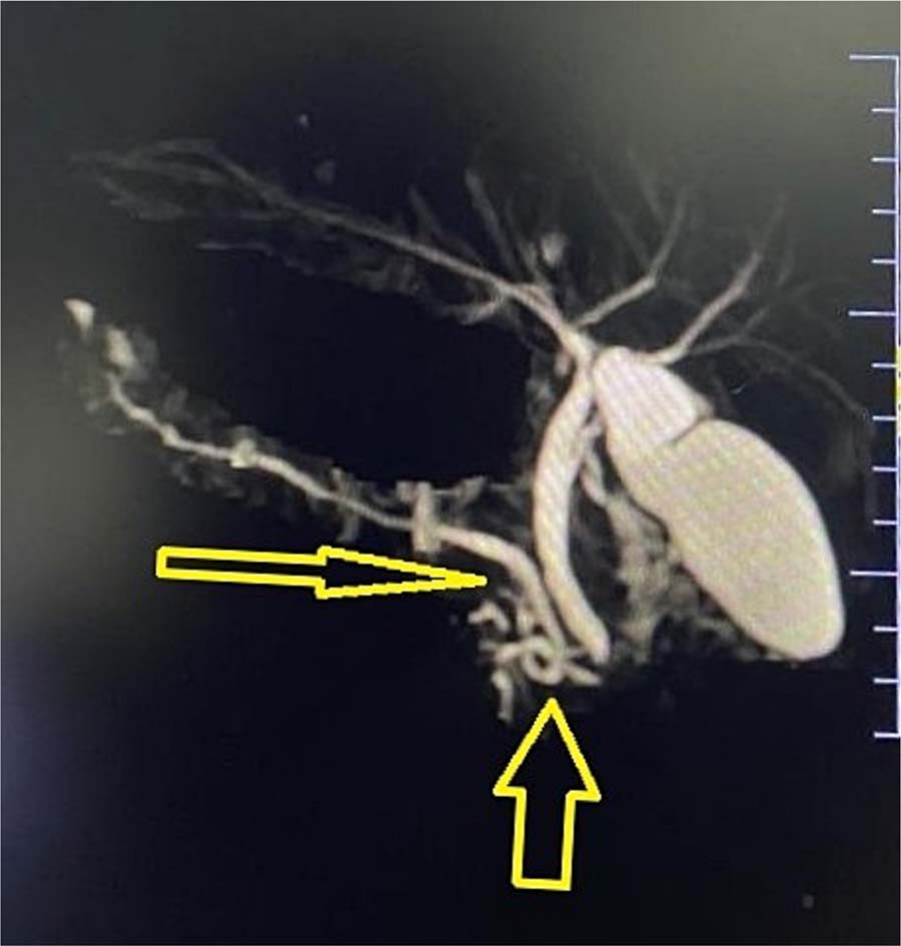
MRCP showing abnormal pancreatic duct anomaly.

Imaging from an ultrasound indicated the main pancreatic duct was dilated to 4 mm, and a computed tomography (CT) scan confirmed dilated pancreatic duct with a Modified CT Severity Index of 4 (Fig. 1). A magnetic resonance cholangiopancreatography (MRCP) revealed a Type 3B biliary duct variation and ansa pancreatica, a looping duct connecting the main and accessory ducts draining via the minor papilla (Fig. 2).

This led to the diagnosis of acute-on-chronic pancreatitis, secondary to ansa pancreatica. The patient, initially managed conservatively, underwent Frey’s procedure through a right subcostal incision with left extension. Frey’s procedure was performed along with ductal drainage and pancreatic head decompression, as the looping duct in the pancreatic head precluded adequate treatment via endoscopic retrograde cholangiopancreatography (ERCP) or pancreatic duct papillectomy. This approach was also necessitated by the unavailability of a pediatric expert pediatric gastrosurgeon at our center. The patient was managed with fluids, analgesics, and antibiotics post-surgery, and drain removal was also done on day 5 after ensuring no pancreatic leakage. Patient remained pain-free, with normalized amylase, lipase levels, and weight gain was noted on subsequent follow-ups.

## Discussion

A wide number of ductal anatomy is encountered in the pancreas, ansa pancreatica being one of them. Ansa pancreatica is a developmental abnormality in which the accessory pancreatic duct is blocked and substituted by a loop originating from the main duct, and has a prevalence of 1.1% [7, 8].

It is considered to be clinically significant due to its association with acute pancreatitis. The most frequent causes of pancreatitis include gallstones, alcohol consumption, and metabolic issues. Nevertheless, differences in pancreatic duct structure, like pancreas divisum, annular pancreas, and ansa pancreatica, are uncommon causes of acute pancreatitis [9]. ARP has around a 36% chance of converting to chronic pancreatitis, which is significantly higher in smokers and alcoholics. Our patient showed dilated pancreatic duct, highlighting progression. [10]

Adibelli *et al.* found that individuals experiencing recurrent pancreatitis exhibited a greater prevalence of ansa pancreatica compared to the general population [11]. Recent evidence suggests that the curved duct causes stasis and, hence, irritation and inflammation [8]. Recurrent pancreatitis is uncommon in younger age group, contrary to our case.

Majority of the cases of ansa pancreatica are diagnosed incidentally and are diagnosed either postoperatively, cadaverically, or radiologically in patients presenting with acute pancreatitis, and thus radiographical imaging like MRCP is vital in accurately diagnosing the pathology. Technical challenges arise when navigating the minor papilla in patients with ansa pancreatica, as this specific anatomical variant hinders successful endoscopic cannulation [9, 11, 12]. However, in cases of failed sphincterotomy, endoscopic ligation of the ansa deformity has also provided good results [7].

Frey’s involves from an anterior approach, a lateral incision be made on the pancreatic duct, and a side-to-side anastomosis then be made between the pancreas and a mobilized loop of jejunum, removing the diseased head of the pancreas wherein to get a clear view of the main pancreatic duct allowing better drainage [13].

A study reported 92% of the patients were pain-free at median 17 months with <12% having perioperative complications after Frey’s procedure for chronic pancreatitis [14].

While endoscopic interventions like minor papilla sphincterotomy are often first-line therapies for managing ductal anomalies, our case demonstrates the potential utility of Frey’s procedure in addressing pancreatitis in the context of a persistent ductal deformity.

## Conclusion

Ansa pancreatica is one of the causes of recurrent pancreatitis in young patients without alcohol use history. The diagnosis, confirmed by MRCP, shows the importance of considering such anatomical ductal variants as underlying causes in patients with recurrent pancreatitis. Our patient underwent Frey’s procedure with excellent postoperative outcome and clinical improvements showing surgical management as a potential option. Recognition of such anatomical variants early is important to plan optimal intervention strategies especially in a country with limited resources.
